# Osmotic regulation of UT‐B urea transporters in the RT4 human urothelial cell line

**DOI:** 10.14814/phy2.14314

**Published:** 2019-12-23

**Authors:** Alan Farrell, Gavin Stewart

**Affiliations:** ^1^ School of Biology & Environmental Science Science Centre West University College Dublin Dublin 4 Ireland

**Keywords:** osmolality, urea, urothelial, UT‐B

## Abstract

Facilitative UT‐B urea transporters play important physiological roles in numerous tissues, including the urino‐genital tract. Previous studies have shown that urothelial UT‐B transporters are crucial to bladder function in a variety of mammalian species. Using the RT4 bladder urothelial cell line, this study investigated the potential osmotic regulation of human UT‐B transporters. Initial end‐point PCR experiments confirmed expression of both UT‐B1 and UT‐B2 transcripts in RT4 cells. Western blotting analysis revealed glycosylated UT‐B protein to be highly abundant and immunolocalization experiments showed it was predominantly located on the plasma membrane. Further PCR experiments suggested that a 48 hr, NaCl‐induced raise in external osmolality increased expression of UT‐B transcripts. Importantly, these NaCl‐induced changes also significantly increased UT‐B protein abundance (*p* < .01, *n* = 7, ANOVA), whereas mannitol‐induced changes in external osmolality had no effect (NS, *n* = 4, ANOVA). Finally, similar increases in both UT‐B RNA expression and protein abundance were observed with urea‐induced changes to external osmolality (*p* < .05, *n* = 4, ANOVA). In conclusion, these findings strongly suggest that increases in external osmolality, via either NaCl or urea, can regulate human urothelial UT‐B transporters.

## Graphical abstract

Facilitative UT‐B urea transporters play a critical role in protecting the bladder urothelial cells from damage caused by urea. Using the RT4 cell line, this study shows that increases in external osmolality (via either NaCl or urea, but not mannitol) significantly increase UT‐B protein abundance. This osmotic regulation of UT‐B transporters may be of clinical importance and requires further investigation.

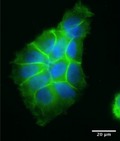

## 1 INTRODUCTION

Facilitative urea transporters allow rapid movement of urea across plasma cell membranes and, in mammals, are encoded by two genes—Slc14a1 (UT‐B) (Olives et al., 1994) and Slc14a2 (UT‐A) (You et al., 1993). These transporters play a vital role in a number of physiological processes, including the urinary concentrating mechanism (Fenton, Chou, Stewart, Smith, & Knepper, 2004; Yang, Bankir, Gillespie, Epstein, & Verkman, 2002). While UT‐A transporters are predominantly located within the kidney, UT‐B transporters are found in tissues throughout the urino‐genital tract—including kidney, ureter, bladder, and urethra (Lucien et al., 2005).

UT‐B transporter protein has been detected in the bladder urothelial cell layers of various mammalian species—including rats (Spector, Yang, Liu, & Wade, 2004), mice (Lucien et al., 2005), dogs (Spector, Yang, & Wade, 2007) and humans (Walpole, Farrell, McGrane, & Stewart, 2014). Crucially, it has been shown that UT‐B knockout mice suffer DNA damage and apoptosis in the bladder, illustrating that UT‐B plays a critical physiological role in protecting bladder urothelium (Dong et al., 2013). In addition, it was also shown that urea levels were significantly higher in UT‐B null urothelium compared to wild type, indicating that UT‐B helps remove toxic intracellular urea from bladder urothelial cells (Dong et al., 2013).

Importantly, although no link has been reported between UT‐B and human kidney disease (Capriolli, Visentainer, & Sell, 2017), UT‐B allelic variation has been shown to affect bladder cancer risk levels (Garcia‐Closas et al., 2011; Rafnar et al., 2011). From the physiological aspect, it has also been reported that there is a direct association between UT‐B and final voided urine concentration, as measured by urinary specific gravity (Koutros, Baris, Fischer, Tang, & Garcia‐Closas, 2013). More recently, UT‐B expression has also been shown to be downregulated in bladder urothelial cancer (Hou, Alemozaffar, et al., 2017a; Li et al., 2014) and a mutated UT‐B transporter has been reported in this disease (Hou, Alemozaffar, et al., 2017a). Understanding the physiological regulation of human urothelial UT‐B transporters is therefore of potential clinical significance.

Relatively little is understood regarding how UT‐B urea transporters are regulated, certainly in comparison to renal UT‐A transporters (Stewart, 2011). However, investigations of urino‐genital tract UT‐B proteins have previously shown that hydration status can affect levels of transporter abundance. For example, changes in UT‐B1 have been reported in mouse urino‐gential tissues, where two days of dehydration significantly reduced UT‐B1 abundance in both bladder and ureter (Lucien et al., 2005). In direct contrast, water restriction in rats actually increased UT‐B1 abundance in rat ureter (Spector et al., 2004). Importantly, further functional studies in rats confirmed that (i) urea was reabsorbed from the urine while it was stored in the rat bladder, and (ii) that hydration status altered these urea reabsorption rates (i.e., physiological regulation occurs) (Spector, Deng, & Stewart, 2011). However, there have been no detailed studies investigating the effect of hydration status on the abundance of UT‐B protein in human urothelial cells.

Our previous studies reported that glycosylated UT‐B transporter protein is located in the umbrella cells of the human bladder urothelial layer (Walpole et al., 2014). The aim of this current study was therefore to use the human RT4 bladder urothelial cell line to investigate the effect of increases in external osmolality upon the expression and abundance of human UT‐B urea transporters.

## 2 METHODS

### 2.1 Tissue culture

Human epithelial bladder transitional papilloma RT4 cells (ATCC, Manassas, VA) were cultured in McCoy's 5A modified medium (306 ± 12 mOsm, *N* = 3), supplemented with 10% heat inactivated fetal bovine serum. Once cells reached 90% confluency, they were sub‐cultured in a 1:10 dilution. Media were removed and replaced with fresh media every 2 days. Cells were incubated at 37°C with 5% CO_2_. For regulatory experiments, cells were exposed to media containing additional NaCl, mannitol or urea for 48hr prior to RNA extraction or protein isolation. Osmolalities of experimental media were confirmed using an Osmomat 030 osmometer (Gonotec, Germany).

### 2.2 RNA preparation

RT4 cells were grown in 75cm^2^ polycarbonate flasks to 85% confluency. Total RNA was extracted using a standard RNA isolation protocol employing RNA Stat (AMS Biotechnology, Abingdon, UK), BCP (1‐Bromo‐3‐chloropropane), isopropanol, and ethanol. Total RNA samples were treated with DNAse enzyme for 25 min at 37°C before being quantified using a ND‐1000 Nanodrop spectrophotometer (Labtech International, Batam, Indonesia). Samples were stored at −80°C until use.

### 2.3 End‐point PCR

cDNA was prepared using a SensiFast cDNA kit (Bioline, London, UK) and employing either human bladder total RNA (AMS Biotechnology, UK) or prepared RT4 total RNA. PCR amplification was carried out using these cDNA samples together with *Go‐Taq* polymerase enzyme (Promega, Kilkenny, Ireland), using UT‐B, AQP3, AQP7, AQP9, NaKATP or Actin primers (see Table 1). Initial denaturation at 94°C for 2 min was followed by 30 or 35 cycles at 94°C for 30 s, 55°C or 60°C for 30 s, and 72°C for 30 s. Final extension was at 72°C for 5 min.

**Table 1:** Summary table of all end‐point PCR primers

Primer Set	Forward	Reverse	Target	Size (bp)
F1/R5	5‐ GCCAGGAAGCCAGCTAGAGTGGTC−3	5‐CTGTCCTGGCTGAGCAAGAGG−3	UT‐B1 UT‐B2	608 450
F3/R5	5‐CTAGGGCACACGTCATGCTGATTC−3	5‐CTGTCCTGGCTGAGCAAGAGG−3	UT‐B2	574
F4/R5	5‐CATGAAAGAACTTGCCAACCAGCTTAAAG−3	5‐ CTGTCCTGGCTGAGCAAGAGG−3	UT‐B	219
F6/R10	5‐GGTGGGAGTACTCATGGCTGTCTTTTC−3	5‐GCCATAAAGTTTGCCATGCCG−3	UT‐B	609
AQP3	5‐GGGAGCCTTCTTGGG TGCTG−3	5‐GGAGGTGCCAATGACCAGGAC−3	AQP3	286
AQP7	5‐GAGGAAGATGGT GCGAGAGTTC−3	5‐GAAGGAGCCCAGGAACTGCC−3	AQP7	283
AQP9	5‐GCTCCGTATCTATCTATCTCTGGC−3	5‐ CAACCAAAGGGCCCACTACAG−3	AQP9	304
NaKATP	5‐CAGCAGAAGCTCATCATTGTGGA−3	5‐GTTCTTCATGCCCTGCTGGAAGA−3	NaKATP	758
Actin	5‐GTGCTGTCTGGCGGCACCACCAT−3	5‐CCTGTAACAACGCATCTCATAT−3	Actin	514

Note

All sequences of both forward and reverse primers used within the PCR experiments, including precise target and exact size (in bp) of the expected product.

### 2.4 Protein preparation

RT4 cells were grown to 85% confluency in 75cm^2^ polycarbonate flasks, washed twice in homogenization buffer (300mM Mannitol, 12mM HEPES, pH 7.6), before physical detachment using a disposable cell scraper. The RT4 cell suspension was homogenized with a Polytron PT1200 homogenizer (Kinematica, Switzerland). The lysate was centrifuged at 1000*g* for 5 min at 4°C. The supernatant was further centrifuged at 17,000*g* for 25 min at 4°C. The resulting pellets were re‐suspended in homogenization buffer for use as a membrane‐enriched protein fraction. An aliquot of the supernatant was retained to represent a cytosol‐enriched protein fraction.

### 2.5 Antibodies

UT‐B proteins were detected using the previously characterized hUT‐Bc19 antibody (Walpole et al., 2014), which had been raised against a 19 amino acid peptide (NH_2_‐EENRIFYLQAKKRMVESPL‐COOH) corresponding to the C‐terminal end of human UT‐B1. Commercially available antibodies for AQP3 (SAB5200111, Sigma‐Aldrich), NaKATP (sc‐28800, Santa Cruz Biotechnology) and GAPDH (sc‐66163, Santa Cruz Biotechnology) were also used, together with horseradish peroxidase conjugated secondary anti‐rabbit IgG antibody (65–6120, Invitrogen) or anti‐mouse IgG antibody (61–6520, Invitrogen).

### 2.6 Immunoblot analysis

All protein samples were mixed at a 1:1 ratio with 2X Laemmli buffer and heated at 70°C for 10 min before being loaded (~5–10 μg per lane) on an 8 to 16% TGX gel for SDS‐PAGE. The separated proteins were transferred to a nitrocellulose membrane and incubated with primary antibody for 16 hr at room temperature, in either 1:5,000 hUT‐Bc19, 1:1,000 GAPDH, 1:1,000 AQP3 or 1:500 NaKATP. Blots were washed and then incubated for 1 hr with 1:5,000 anti‐rabbit or anti‐mouse antibody conjugated to horseradish peroxidase. After further washing the proteins were imaged using Western Lighting Plus ECL reagents (Perkin Elmer, USA) and a LAS4000 Imager (Fujifilm, Japan). For deglycosylation experiments protein samples were incubated with and without peptide‐*N*‐glycosidase F enzyme for 2 hr at 37°C. For peptide incubation experiments hUT‐Bc19 was pre‐incubated with specific or non‐specific peptide for 24 hr using a rotating mixer.

### 2.7 Immunofluorescent localization

RT4 cells, grown on glass cover slips for 72 hr, were fixed with 4.0% paraformaldehyde for 30 min. and then permeabilized with Triton X‐100 for 20 min. Cells were quenched with 30 mM glycine for 30 min before incubation for 2 hr in 1:400 hUT‐Bc19 antibody. Following incubation for 1 hr in goat anti‐rabbit antibody conjugated to Alexafluor 488 (1:500), the cells were incubated in Hoechst 33,342 nucleic acid stain (1:2000) for 10 min. Cover slips were mounted on glass slides using Dako mounting medium.

## 3 RESULTS

**Figure 1:**
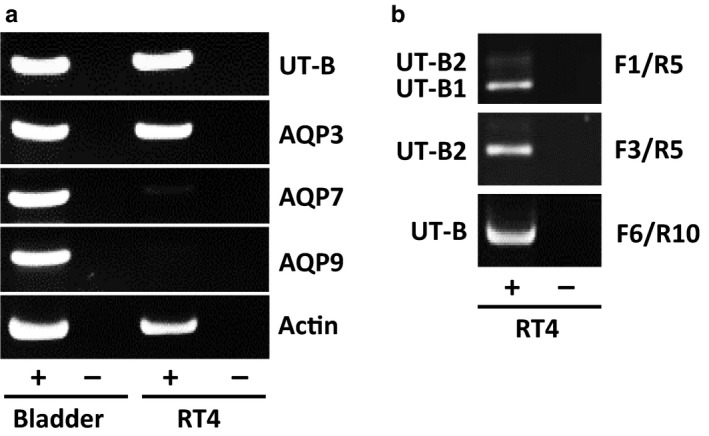
End‐point RT‐PCR experiments showing UT‐B1 is the main UT‐B transcript present in the RT4 human urothelial cell line. (A) End‐point RT‐PCR experiments using a variety of primers showed that RT4 cells strongly expressed both UT‐B (F4/R5) and AQP3, but not AQP7 or AQP9. In contrast, human bladder was confirmed to highly express UT‐B, AQP3, AQP7, and AQP9. (B) Further experiments revealed that UT‐B1 was the predominant transcript in RT4 cells. Isoform‐specific primers (F1/R5) mainly detected UT‐B1, rather than UT‐B2. However, some UT‐B2 expression did occur, as confirmed through a set of UT‐B2‐specific primers (F3/R5). **Key:** **+** = reverse transcriptase present; **-** = reverse transcriptase absent

Initial end‐point PCR experiments revealed that RT4 urothelial cells strongly expressed both UT‐B and AQP3, but not AQP7 or AQP9 (Figure 1a). Using F1/R5 UT‐B primers, it was revealed that UT‐B1 was the predominant transcript, with UT‐B2 expressed at a lower level (Figure 1b). Expression of UT‐B2 was confirmed using the UT‐B2‐specific F3/R5 primer set (Figure 1b). Sequencing of PCR products (data not shown) revealed RT4 cells to possess the Jk(A) allelic variation of UT‐B.

**Figure 2:**
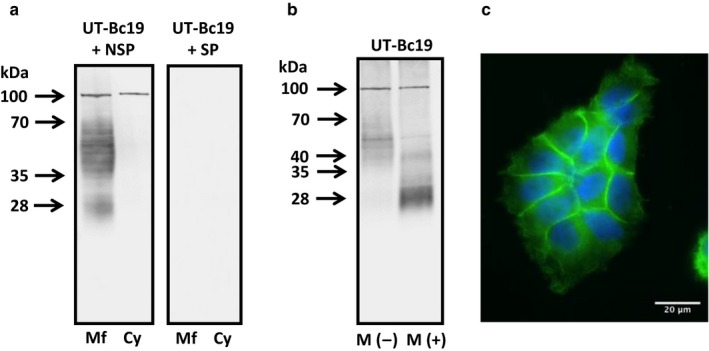
Western blotting and immunolocalization experiments suggesting glycosylated UT‐B protein is abundant in the plasma membrane of RT4 human urothelial cells. (a) Using hUTBc19 pre‐incubated in non‐specific peptide, strong 28 and 35–70 kDa signals were detected in membrane‐enriched but not cytosol‐enriched protein. In contrast, using hUTBc19 pre‐incubated in the specific, immunizing peptide, no such signals were detected. (b) Deglycosylation treatment with PNGaseF enzyme reduced the 35–70 kDa UT‐B signal to a strong 28 kDa band and a weaker 40 kDa band. (c) Immunolocalization experiments revealed UT‐B protein to be predominantly found in the plasma membrane of the RT4 cells. **Key: Mf** = membrane‐enriched protein; **Cy** = cytosol‐enriched protein; ***M*(‐)** = untreated membrane‐enriched protein (i.e., buffers only); ***M*(+)** = PNGaseF treated membrane‐enriched protein (i.e., buffers + enzyme)

Using the previously characterized hUTBc19 antibody, pre‐incubated in a non‐specific peptide, western blotting analysis showed strong signals for UT‐B protein at 28 and 35–70 kDa in RT4 membrane‐enriched protein samples, but not in cytoplasmic‐enriched samples (Figure 2a). In contrast, pre‐incubation with the same amount of the specific, immunizing peptide completely ablated all signals (Figure 2a). [NOTE: Although these findings did not conclusively prove that all signals were UT‐B, they did confirm that all signals were due to the hUTBc19 antibodies and not due to any contaminant]. Deglycosylation treatment with PNGaseF enzyme shifted the 35–70 kDa smeared signal in membrane‐enriched protein to a strong distinct band at 28 kDa, plus a weaker signal at 40 kDa (Figure 2b). Immunolocalization experiments then revealed that a strong UT‐B signal in RT4 cells was indeed located in the plasma membrane, with only weak intracellular staining observed (Figure 2c). Interestingly, there was little UT‐B detected in the outermost plasma membrane surfaces of RT4 cell clusters (Figure 2c).

**Figure 3:**
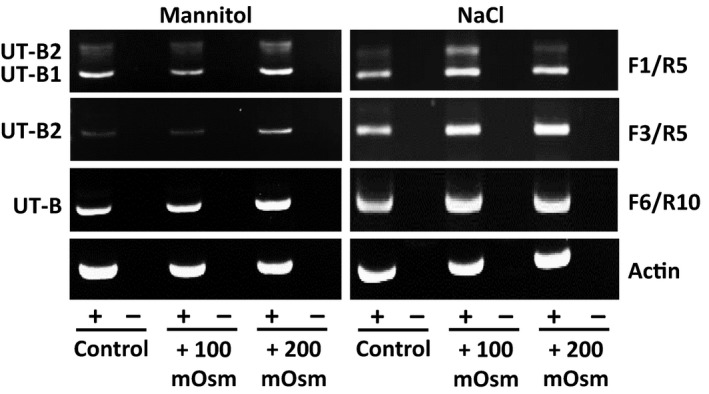
End‐point RT‐PCR experiments showing effect of mannitol and NaCl‐induced changes in external osmolality on UT‐B expression. Mannitol‐induced increases in osmolality appeared to have little or variable effect on UT‐B expression, except perhaps for small increases in F3/R5 (i.e., UT‐B2) and F6/R10 (i.e., general UT‐B) signals with +200 mOsm. In contrast, NaCl‐induced changes appeared to increase all UT‐B signals (i.e., UT‐B1, UT‐B2 and general UT‐B) in both +100 mOsm and +200 mOsm samples. Finally, there were no changes in actin expression throughout all experiments. [NOTE: Data shown are representative of two experiments performed and is therefore not quantitative.]

To investigate effects of changes in external osmolality on UT‐B transporters, cells were incubated for 48 hr in media containing various levels of mannitol or NaCl, to raise external osmolality by 100 and 200 mOsm. Initial end‐point PCR experiments suggested that mannitol‐induced changes in osmolality had little or variable effect on UT‐B expression (Figure 3). In contrast, more consistent increases in the expression of UT‐B1 and UT‐B2 were observed with NaCl‐induced changes in osmolality (Figure 3).

**Figure 4:**
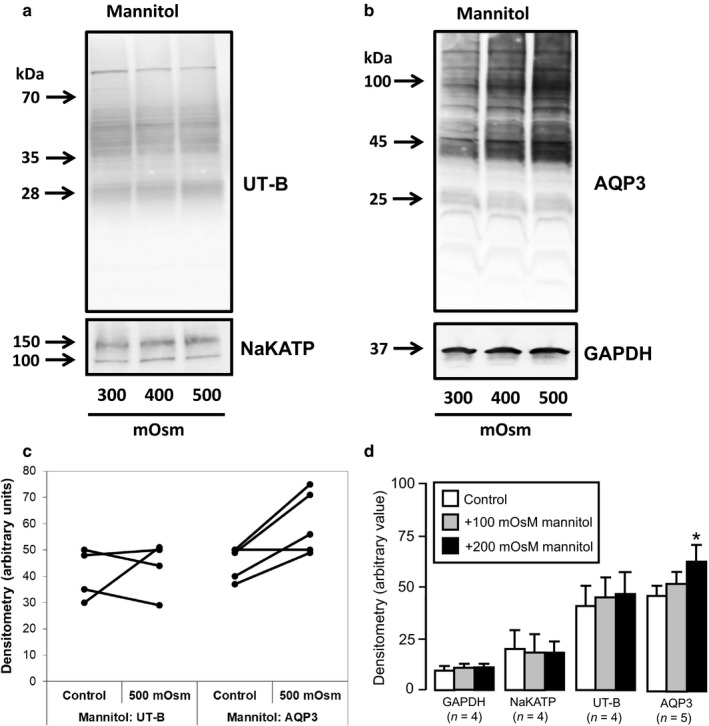
Western blotting experiments showing effects of mannitol‐induced increases in external osmolality on protein abundance. (a) No changes in 28 or 35–70 kDa UT‐B signals were observed with mannitol treatment. In addition, there was no change in 100 and 150 kDa NaKATP protein signals. (b) Significant increases in 45 and 100 kDa AQP3 protein signals were detected with both +100 and +200 mOsm mannitol, while no such effect was observed for 37 kDa GAPDH. (c) Summary graph comparing the densitometry values in control (~300 mOsm) to + 200 mOsm mannitol (~500 mOsm) treatments in all experiments for both UT‐B (*n* = 4) and AQP3 (*n* = 5). (d) Bar graphs illustrating mean densitometry values for GAPDH, NaKATP, UT‐B and AQP3 after +100 and +200 mOsm mannitol‐induced treatments. **Key:** * = *p* < .05, ANOVA

**Figure 5:**
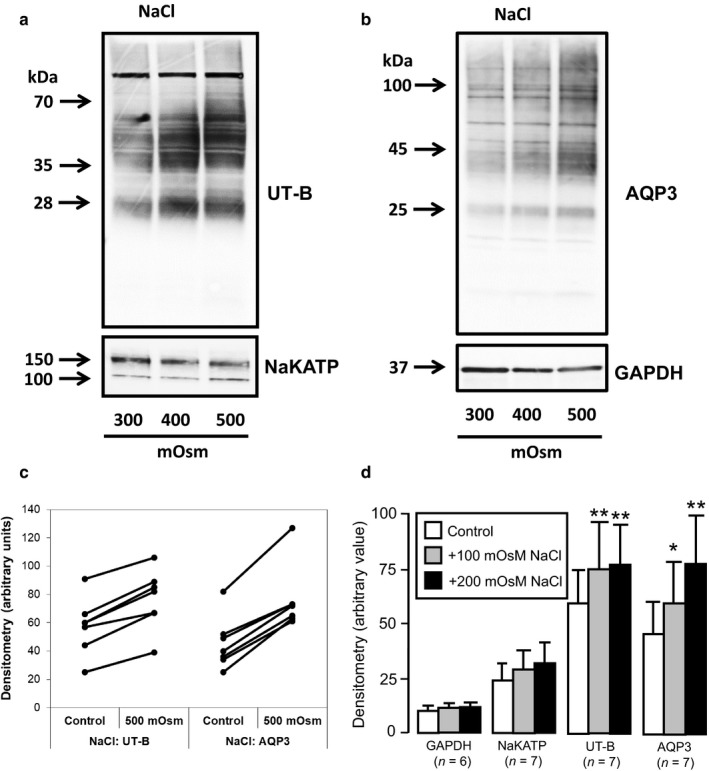
Western blotting experiments showing effects of NaCl‐induced increases in external osmolality on protein abundance. (a) Increases in both 28 and 35–70 kDa UT‐B signals were observed with NaCl treatment, whereas there was no change in NaKATP signals. (b) Significant increases were also observed in 45 and 100 kDa AQP3 protein, with no effect observed for 37 kDa GAPDH. (c) Summary graph comparing the densitometry values in control (~300 mOsm) to +200 mOsm NaCl (~500 mOsm) treatments in all experiments for UT‐B (*n* = 7) and AQP3 (*n* = 7). (d) Bar graphs illustrating mean densitometry values for GAPDH, NaKATP, UT‐B and AQP3 after +100 and +200 mOsm NaCl‐induced treatments. **Key:** * = *p* < .05, ANOVA; ** = *p* < .01, ANOVA

Next, western analysis was performed on mannitol‐treated cells and suggested no change in UT‐B protein abundance occurred within RT4 plasma membranes (Figure 4a), but that there was an increase in AQP3 transporters (Figure 4b). This increase in AQP3 was mainly seen in the ~45 kDa (dimer) and ~100 kDa (tetramer) signals, rather than the ~25 kDa (monomer) signal. These patterns of response were generally repeated (Figure 4c) and densitometry analysis confirmed there was no change in UT‐B (NS, *N* = 4, ANOVA), but that mannitol treatment significantly increased levels of AQP3 protein (*p* < .05, *N* = 5, ANOVA) (Figure 4d). Western analysis was then performed on NaCl‐treated cells and showed that this time there were increases in both UT‐B (Figure 5a) and AQP3 (Figure 5b) protein abundance, although NaKATP and GAPDH were unaffected. While initial levels of protein abundance in control conditions varied from experiment to experiment, there were very consistent, similar sized increases for UT‐B and AQP3 (Figure 5c). Densitometry analysis confirmed significant changes for both UT‐B (*p* < .01, *N* = 7, ANOVA) and AQP3 (*p* < .01, *N* = 7, ANOVA) (Figure 5d). For example, the mean (± S.D.) densitometry values for UT‐B went from 58 ± 20 in control media (~300 mOsm) to 75 ± 21 in + 200 mOsm NaCl‐treated cells (~500 mOsm), an increase of ~ 30%. Finally, it should be noted that while 48 hr exposure to NaCl‐induced increased UT‐B, exposures of 6 hr or 24 hr had less effect (data not shown).

**Figure 6:**
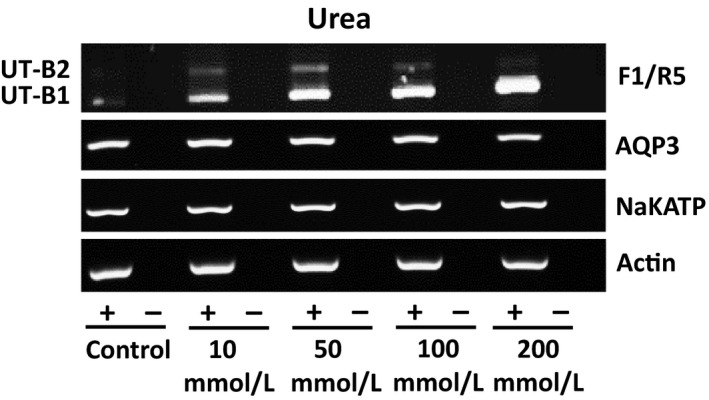
End‐point RT‐PCR experiments showing urea‐induced changes in external osmolality increase UT‐B RNA expression. Urea‐induced increases in osmolality appeared to increase both UT‐B1 and UT‐B2 expression, with the greatest increase observed in UT‐B1 with +200 mM urea. In contrast, no such increase was seen for AQP3, NaKATP, or actin. [Data shown are representative of two experiments performed and is therefore not quantitative.]

**Figure 7:**
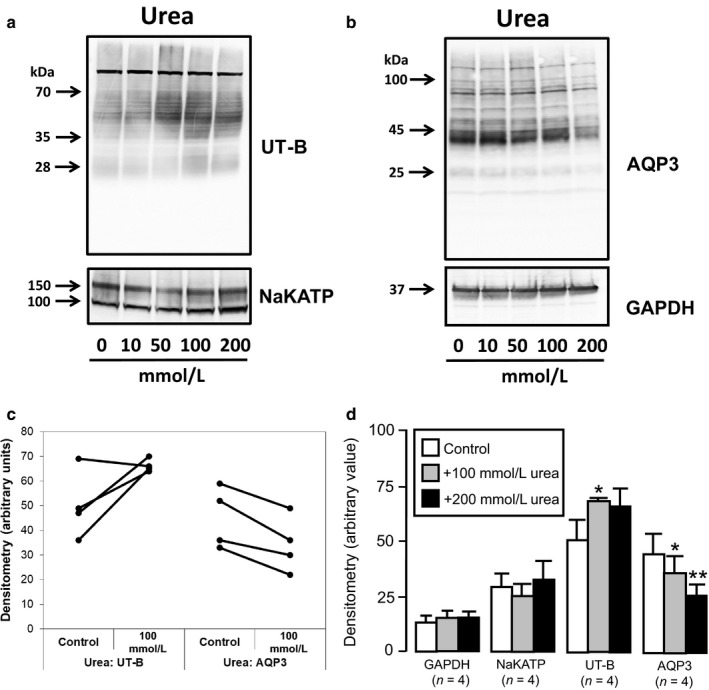
Western blotting experiments showing effects of urea‐induced increases in external osmolality on protein abundance. (a) Increases in 28 or 35–70 kDa UT‐B signals were observed with +50, +100, and +200 mM urea treatment, while there was no change in NaKATP protein. (b) In direct contrast, significant decreases in 45 kDa AQP3 protein signals were observed with 50, 100, and 200 mM urea. Finally, no such changes were detected for GAPDH protein. (c) Summary graph comparing the densitometry values in control to +100 mM urea treatments in all experiments for both UT‐B (*n* = 4) and AQP3 (*n* = 4). (d) Bar graphs illustrating mean densitometry values for GAPDH, NaKATP, UT‐B, and AQP3 after +100 and +200 mM urea‐induced treatments. **Key:** * = *p* < .05, ANOVA: ** = *p* < .01, ANOVA

Finally, the effects of 48 hr exposure to various levels of external urea on RT4 cells were investigated. Using a range of 0 to 200 mM urea, end‐point PCR experiments suggested that urea stimulated expression of UT‐B, and particularly the UT‐B1 transcript, but had little effect on AQP3, NaKATP, or actin (Figure 6). Importantly, further investigation confirmed that external urea exposure increased UT‐B protein abundance (Figure 7a), while it actually decreased the levels of AQP3 transporters (Figure 7b). Additional experiments generally showed the same patterns (Figure 7c) and densitometry analysis confirmed that 100 mM urea treatment increased UT‐B protein abundance (*p* < .05, *N* = 4, ANOVA), decreased AQP3 (*p* < .05, *N* = 4, ANOVA) and had no effect on either NaKATP or GAPDH (NS, *N* = 4, ANOVA) (Figure 7d). For example, the mean (± S.D.) densitometry values for UT‐B went from 50 ± 14 in control media (~300 mOsm) to 66 ± 2 in + 100 mM urea‐treated cells (~400 mOsm), an increase of ~30%. Lastly, the effects on protein abundance of treatments with mannitol, NaCl, and urea were summarized (Table 2).

**Table 2:** Summary of changes in protein abundance produced by alterations in external osmolality induced by mannitol, NaCl, and urea

	Mannitol	NaCl	Urea
UT‐B	‐	↑	↑
AQP3	↑	↑	↓
NaKATP	‐	‐	‐
GAPDH	‐	‐	‐

## 4 DISCUSSION

Our initial studies confirmed that the RT4 cell line strongly expressed UT‐B at both the RNA (Figure 1a) and protein level (Figure 2a). The predominant RNA transcript was UT‐B1 (Figure 1b), while both 28 kDa unglycosylated and 35–70 kDa glycosylated proteins were present (Figure 2b). Immunolocalization studies then confirmed UT‐B protein to be located on the plasma membrane (Figure 2c). These general findings closely agree with our previous report on human bladder UT‐B transporters (Walpole et al., 2014). In further agreement with the literature (Rubenwolf et al., 2014), these RT4 cells also abundantly expressed AQP3 transporters and appeared to be an appropriate model for our investigations. Interestingly, derived from urinary bladder transitional cell papilloma, we found that RT4 cells possess the Jk(A) allelic variation of UT‐B, reported to confer an increased risk for bladder cancer (Garcia‐Closas et al., 2011; Rafnar et al., 2011). However, the RT4 cells did not contain the 24 nucleotide in‐frame, exon 4 deletion mutant recently reported in bladder cancer cells (Hou, Alemozaffar, et al., 2017a).

The precise nature of the UT‐B transporters in the mammalian bladder remains unknown. Previous studies have reported similar‐sized, unglycosylated UT‐B bladder proteins at 29 kDa in mice (Lucien et al., 2005), 32 kDa in rats (Spector et al., 2004) and 30 kDa in humans (Walpole et al., 2014). Similarly, in this current study, we were unable to clearly determine the exact UT‐B proteins present. As UT‐B1 was the main transcript, it would be predicted that the resulting unglycosylated UT‐B1 protein would be ~40 kDa in size. Although deglycosylation experiments did reveal a weak 40 kDa signal, there was a much stronger unglycosylated protein revealed at ~28 kDa (Figure 2b). We have previously suggested, due to our UT‐B antibodies being targeted to the C‐terminal, that the 30 kDa UT‐B protein found in human bladder is most likely the result of a N‐terminal truncation event (Walpole et al., 2014). Alternatively, the recent study by Hou et al. overexpressed both UT‐B isoforms in HEK293 cells and showed unglycosylated proteins at ~38 kDa for UT‐B2 and ~30 kDa for UT‐B1 (Hou, Alemozaffar, et al., 2017a). It is therefore also feasible that what we have observed in RT4 cells is UT‐B2 protein at a low abundance (~40 kDa) and UT‐B1 protein at high abundance (at ~28 kDa), which would match the relative RNA expression we detected (Figure 1b). Unfortunately, we currently do not possess reliable UT‐B N‐terminal antibodies to confirm which, if either, of these suggestions is correct.

Studies investigating mammalian urothelial UT‐B transporter expression and abundance have generally suggested that dehydration can have significant effects. For example, dehydration increased UT‐B protein abundance in both rat kidney (Lim et al., 2006) and rat ureter (Spector et al., 2004). Interestingly, while Spector et al. reported a 49% increase in ureter, there was only a marginal 14% increase in bladder, where UT‐B was already highly abundant in control conditions (Spector et al., 2004). Within our current study, increasing external media osmolality with mannitol had no significant effect on UT‐B abundance, but did significantly increase AQP3 transporters (Figure 4). In direct contrast, NaCl‐induced changes in external media did stimulate increased UT‐B protein abundance (Figure 5). As expected, NaCl exposure also increased AQP3 transporters, as has been previously reported, for example in the NHU cell line (Rubenwolf, Georgopoulos, Kirkwood, Baker, & Southgate, 2012). Lastly, treatment with 100 mM urea also significantly increased UT‐B protein abundance (Figure 7). Unlike with previous treatments, urea exposure actually significantly decreased AQP3 transporter protein (Figure 7), with the lack of a urea‐induced increase in this urothelial aquagylceroporin again agreeing with a previous study (Rubenwolf et al., 2012). Overall, whilst NaCl and urea upregulated UT‐B transporters, only NaCl (or mannitol) upregulated AQP3 (Table 2).

So, what is the physiological relevance of urea transporter regulation in bladder urothelial cells? The classical view that the mammalian bladder is a simple storage vessel, which along with the ureter has no transport capabilities, is no longer consistent with the research literature. For example, it is now known that the composition of urine actually changes as it passes along the human urinary tract (Cahill, Fry, & Foxall, 2003). More specifically, it has long been known that urea can pass across the human bladder urothelial layers (Lilly & Parsons, 1990). Importantly, other studies with rat bladder have already shown that hydration status directly alters urea transport rates (Spector et al., 2011), presumably at least partly through changes in UT‐B transporters. Our results suggest that the levels of human urothelial UT‐B are also regulated by changes in the urine osmolality. Hence, concentrated urine containing higher levels of urea would stimulate an increase in UT‐B transporters in bladder umbrella cells, therefore facilitating the rapid removal of the toxic urea that would be passing into the urothelial lining in greater amounts.

In this current study, the significant 30% increase in UT‐B protein abundance obtained is about twice the size of the effect previously observed in dehydrated rat bladder (Spector et al., 2004). However, the high levels of UT‐B in untreated RT4 bladder cells, while being similar to that reported for human bladder, may hinder further investigations into the exact regulatory mechanisms involved. Instead, we suggest that human ureter derived cell lines may be a better model, since they are likely to see greater osmolality‐induced increases in UT‐B abundance due to low control levels of the transporter—perhaps similar or greater to the ~50% increase in ureter UT‐B seen in dehydrated rats (Spector et al., 2004). The initial step in these future studies would be to first confirm low expression and abundance of UT‐B in the human ureter tissue. Further studies, using a suitable ureter cell line, could then investigate the role of various cellular mechanisms previously shown to be involved in the osmotic regulation of UT‐A urea transporters. For example, elements such as protein kinase C and calcium, which have been shown to be involved in the osmotic regulation of both renal UT‐A1 (Klein, Martin, Kent, & Sands, 2012) and intestinal UT‐A6 (McGrane & Stewart, 2016).

What is the clinical relevance of understanding the regulation of human UT‐B transporters? In the last decade, studies of various cancers have reported a substantial decrease in UT‐B expression in cancerous tissues—including bladder (Hou, Alemozaffar, et al., 2017a; Li et al., 2014), prostate (Vaarala, Hirvikoski, Kauppila, & Paavonen, 2012) and lung (Frullanti et al., 2012). Indeed, in their recent review Hou et al. suggested that UT‐B transporters could therefore be a novel target for cancer research (Hou, Kong, Yang, Xie, & Chen, 2017b). More recently, following initial reports of UT‐B knockout mice suffering depression‐like behavior (Li et al., 2012), UT‐B transporters have also been implicated in various human brain diseases. For example, recent reports have linked UT‐B to Huntington's (Handley et al., 2017), Alzheimer's (Recabarren & Alarcon, 2017) and Parkinson's disease (Santiago, Bottero, & Potashkin, 2018). Future findings on UT‐B regulation, and particularly the resulting functional activities, could therefore have potential benefits to a wide range of human diseases.

In conclusion, our data have shown that UT‐B urea transporters are highly abundant in the RT4 human urothelial cell line. Chronic increases in external osmolality, either through NaCl or urea, lead to a significant increase in glycosylated UT‐B protein abundance. These findings have important implications for our understanding of the physiological regulation of human bladder urothelial transport and further work is required to detail the cellular mechanisms controlling these processes.

## CONFLICT OF INTEREST

There were no conflicts of interest.
